# Need for Comprehensive Hormonal Workup in the Management of Adrenocortical Tumors in Children

**DOI:** 10.4274/jcrpe.1351

**Published:** 2014-06-05

**Authors:** E. Nazlı Gönç, Zeynep Alev Özön, Meltem Didem Çakır, Ayfer Alikaşifoğlu, Nurgün Kandemir

**Affiliations:** 1 Hacettepe University Faculty of Medicine, Department of Pediatric Endocrinology, Ankara, Turkey

**Keywords:** Adrenocortical tumors, adrenal insufficiency, virilization, Cushing’s syndrome, children

## Abstract

**Ob­jec­ti­ve:** Clinical findings do not reflect the excess hormonal status in adrenocortical tumors (ACTs) in children. Identification of abnormal hormone secretion may help provide the tumor marker and delineate those patients with a risk of adrenal suppression following tumor removal. To analyze the impact of complete hormonal assessment regardless of the clinical presentation in hormone-secreting ACTs in childhood.

**Methods:** Association of hormonal workup at diagnosis with the clinical findings and frequency of adrenal suppression postoperatively were analyzed in 18 children with ACT.

**Results:** Seventeen of the 18 patients had functional ACT. Clinical findings suggested isolated virilization and isolated Cushing’s syndrome in 38.8% and 17.6% of patients, respectively. Hormonal workup revealed a frequency of 83.3% for hyperandrogenism. The majority of the tumors (50%) had mixed type hormonal secretion. Hypercortisolism existed in 28.5% of children with isolated virilization and hyperandrogenism was found in 2/3 of children with isolated Cushing’s syndrome. Various androgens other than dehydroepiandrosterone sulfate were also determined to be high in hyperandrogenism. Increased testosterone was a highly prevalent tumor marker. Nine patients (3 with no signs of hypercortisolism) had adrenal suppression following tumor removal which lasted 1-24 months.

**Conclusion:** Complete hormonal workup showed the predominance of mixed hormone-secreting type of tumor in the patients who lacked the appropriate clinical findings and also showed that patients lacking signs of Cushing’s syndrome could have postoperative adrenal suppression. Clinical findings may not reflect the abnormal hormone secretion in all cases and tumor markers as well as risk of postoperative adrenal suppression can best be determined by complete hormonal evaluation at the time of diagnosis.

## INTRODUCTION

Adrenocortical tumors (ACTs) are rare in childhood with an estimated annual incidence of 0.3 cases per million, representing less than 0.2% of all pediatric neoplasms and they account for 6% of all adrenal tumors in children (1). ACTs are functional in most cases and clinical presentation depends on the specific adrenocortical hormones secreted from the tumor. Virilization is the most common presentation, followed by Cushing’s syndrome and hyperaldosteronism. Virilization can be isolated or in combination with hypercortisolism. However, isolated Cushing’s syndrome is rare (2). It has been suggested that secretory abnormalities of the hormones correlate well with clinical symptoms (3), thus the hormonal profile of these patients was not extensively investigated in all cases and generally the function of the tumor was defined according to the clinical presentation. Analysis of hormone profile is crucial for determination of excess hormones as tumor markers as well as recognition of hypercortisolism which may lead to adrenal suppression and ensuing adrenal insufficiency following tumor removal. There is no data in the literature pertaining to frequency and duration of postoperative adrenal suppression in children with ACTs. 

In this retrospective study, we evaluated the hormone profile of children with ACT and analyzed the impact of a detailed hormone workup on diagnosis, preparation for surgery and follow-up. 

## METHODS

The medical records of 18 children with ACT followed in our unit since 1999 were analyzed retrospectively. Cases with adrenal medulla tumors (neuroblastoma, pheochromocytoma and ganglioneuroma) and those with tumors metastatic to the adrenal gland were excluded. Data regarding age, sex, presenting symptoms, period of time from initial symptoms until diagnosis, clinical characteristics, laboratory investigations, imaging, histology, treatment and outcome were extracted from the medical files. Extent of disease at presentation was determined by diagnostic imaging (ultrasound, X-ray, CT, MR imaging). Findings were corroborated by findings at surgery and pathological examination. Tumors were confirmed by histological examination. Since histological criteria to differentiate malignant behavior in childhood ACTs are not reliable (4,5), all lesions were categorized as ACT instead of adenoma or carcinoma. Functionality of the tumor was determined by clinical findings and serum hormone levels, including adrenocorticotropic hormone (ACTH), cortisol, 17-hydroxy progesterone [17(OH)P], androstenedione (AS), dehydroepiandrosterone sulfate (DHEA-S), testosterone (T), estradiol, renin and aldosterone.

Normal serum hormone levels were accepted as <2ng/mL for 17(OH)P; <0.3 ng/mL (prepubertal) and 0.9-2.3 ng/mL (pubertal) for AS; <30 μg/dL (prepubertal) and 30-440 μg/dL (pubertal) for DHEA-S; <20 ng/dL (prepubertal), 20-65 ng/dL (girls, pubertal) and 288-836 ng/dL (boys, pubertal) for T; and 3-18 μg/dL (8.00-9.00 am) for cortisol. 

Virilization was defined as premature or inappropriate pubic or body hair, acne, deepening of voice, enhanced growth, penile enlargement and clitoromegaly. Hyperandrogenism on the other hand, was defined with elevated serum androgen levels regardless of clinical signs of virilization. Findings suggestive of Cushing’s syndrome were rapid increase in weight associated with growth deceleration, presence of moon face, buffalo hump, purple or bright red striae and/or hypertension. Hypercortisolism on the other hand was defined as increased levels of serum cortisol regardless of signs and symptoms of Cushing’s syndrome.

All but three patients had undergone tumor excision. One of the three patients who were accepted as inoperable had hepatic and pulmonary metastases at the time of diagnosis. The second patient had two different kinds of tumor in both liver and adrenal cortex and the third patient had a huge tumor infiltrating the surrounding tissue. All patients who underwent surgery received steroid coverage perioperatively with a maximum duration of three days after the operation. The dose of steroid was 20-30 mg/m2/day prednisolone on the day of operation which was tapered down and withdrawn on the third postoperative day. All patients were assessed for residual tumor and adrenal suppression by evaluation of their hormone profile within one week after cessation of steroid therapy. Adrenal suppression was initially assessed by a fasting, early-morning serum cortisol level. A basal cortisol level of 15 µg/dL or higher ruled out adrenal suppression, whereas a level less than 3 µg/dL suggested adrenal suppression. A level between 3-15 µg/dL required low-dose ACTH test to rule out adrenal suppression. A peak cortisol response of 19.6 µg/dL or more during low-dose ACTH test ruled out adrenal suppression (6). Patients with adrenal suppression were started on hydrocortisone (10-12 µg/m2/d p.o.), were assessed for adrenal glucocorticoid reserve one month after the operation and then every three months to establish recovery from suppression. All patients were followed for tumor recurrence using adrenal hormones as tumor markers periodically. The first two evaluations were carried out one week and one month after the operation and the following - at three-month intervals.

## RESULTS

The ages of the patients (10 girls, 8 boys) ranged from 3 months to 16.1 years at diagnosis and 15 of these 18 children were prepubertal. The ages of the prepubertal group ranged between 3 months and 9 years. The three pubertal cases were 14,16 and 16.1 years of age with a pubertal staging of Tanner 4-5. Duration of symptoms until diagnosis varied between 15 days to 4 years. Seven patients had signs suggestive of virilization alone, three patients had signs suggesting Cushing’s syndrome, five patients had signs suggesting both virilization and Cushing’s syndrome, two patients had abdominal pain (and hypertension in one of them) and the last patient had muscle pain and fatigue associated with hypertension. The clinical characteristics of the patients are shown in Table 1.

All patients were tested for serum electrolytes, androgens, cortisol, renin and aldosterone regardless of the clinical presentation. Seventeen of the 18 (94.4%) patients had functional ACT and 15 of these 17 (88.2%) patients with functional ACT had variable degrees of hyperandrogenism. Only 12 of 15 (80%) patients with hyperandrogenism had clinical signs of virilization. Three of the 7 (42.8%) patients with signs of virilization solely, had laboratory findings suggesting hypercortisolism. Two of these three patients had a basal cortisol level exceeding 30 µg/dL and both had adrenal suppression for 9 and 5 months after tumor removal. The third patient had a basal cortisol level of 14.4 µg/dL, but adrenal suppression persisted for 24 months postoperatively. Five patients had both virilization and clinical signs of Cushing’s syndrome. All but one patient with basal cortisol level of 16.2 

µg/dL had elevated androgen and cortisol levels. Further evaluation of this patient yielded an impaired diurnal rhythm of cortisol as well as elevated 24-hour urinary free cortisol levels. Two of the three patients with isolated Cushing’s syndrome also had elevated androgen levels (Table 2).

One patient (patient #15) had severe hypokalemia (K: 1.89 mEq/L) and hypertension. He was at Tanner stage 4 and the androgen levels were concomitant to his pubertal stage. 

Basal cortisol and ACTH levels were within normal limits. The only hormonal aberration was elevated aldosterone (>1400 pg/mL N: 30-355), low renin [plasma renin activity (PRA): 0.15 

ng/mL/hr; normal: 0.2-1.6] and increased ratio of plasma aldosterone concentration to PRA (aldosterone/PRA >933) suggesting an aldosterone-secreting ACT.

Patient #18 was a 3-year-old girl, presenting with axillary and pubic hair development and progressive abdominal distension. Ultrasound examination demonstrated a right-sided adrenal mass of 5.5x4 cm and multiple solid masses located in the liver. Biopsy specimens taken from both organs revealed two kinds of tumor: hepatoblastoma in the liver and adrenocortical tumor in the adrenal cortex. Androgen levels were also elevated. There were several cases of malignancy in the family history. The first child of the family had died of hepatoblastoma at 2.5 years of age. The mother had a history of breast cancer at the age of 23 and the maternal grandfather had died of colon cancer at the age of 25. 

Patient #9 was a 16-month-old girl with virilization. After the excision of the adrenocortical tumor, all the symptoms and signs disappeared. At 5 years of age, hyperpigmented macules appeared on the oral mucosa and lips and several hamartomatous polyps in the intestine were excised which were histologically confirmed as Peutz-Jeghers polyps. At 5.5 years of age, she was diagnosed to have papillary thyroid carcinoma. Analysis of the STK11 gene showed a missense mutation leading to the amino acid change E199D in the kinase domain, confirming the diagnosis of Peutz-Jeghers syndrome (7).

The single patient with nonfunctional ACT (patient #14) who was at Tanner stage 4 had an adrenal mass 12 cm in diameter. The hormonal workup was completely within normal limits. The presenting finding of hypertension was thought to be due to compression of renal vessels by the tumor mass. 

Thus, in this series, the overall frequency of isolated androgen-secreting ACTs was 27.7% (5/18), that of isolated cortisol-secreting ACTs 5.6% (1/18), that of isolated aldosterone-secreting ACTs 5.6% (1/18) and the frequency of tumors with the capacity to secrete both androgen and cortisol was 55.8% (10/18). The frequency of nonfunctional ACTs was 5.6 % (1/18).

**Androgen Levels in Hyperandrogenism**

Fifteen patients with hyperandrogenism had varying degrees of elevation in androgen levels. Only 9 (60%) had a DHEA-S levels significantly exceeding the upper range of normal. In the remaining 6 (40%) patients, DHEA-S levels were either normal or just at the upper limit of normal. In one of the six patients with low DHEA-S, the only marker of hyperandrogenism was elevated AS (patient #4). Patient #17 had moderately elevated androstenedione, DHEA-S and T levels. The remaining four patients had a T level exceeding 100 ng/dL, a marker for hyperandrogenism (Table 3). 

**Postoperative Adrenal Suppression**

Nine patients had adrenal suppression postoperatively varying from 1-24 months in time. The duration of postoperative adrenal suppression was independent of early-morning cortisol levels as well as of the age of the patient (r=-0.271, p=0.480; r=-0.136, p=0.727, respectively). Three of the 9 patients with postoperative adrenal suppression did not have any sign of Cushing’s syndrome, although two of them had significantly elevated early-morning cortisol levels. Three of the patients with adrenal suppression (two with signs of Cushing - patient #5 and #8 and the other lacking signs of Cushing - patient #3) did not have any elevation in early-morning cortisol levels (Table 2).

**Follow-up**

Fifteen patients had undergone surgical excision of the tumor and 12 of these were tumor-free for 1-11 years after tumor excision. The remaining three patients had more unfavorable outcomes and two were eventually lost to follow-up. The tumor had ruptured during surgery in two of these patients. One (patient #11) of these two patients developed pulmonary and hepatic metastases 5 months after the surgery with serum cortisol, DHEA-S and T levels increasing to even higher levels than that at the time of initial diagnosis. This patient was started on mitotane, cisplatin, etoposide and adriamycin, but was lost to follow-up never coming back after one month of chemotherapy. The other patient (patient #14) had nonfunctional ACT and was started on mitotane and eventually cisplatin, etoposide and radiotherapy following rupture of the tumor during the operation. However, sixteen months later, multiple metastases infiltrating liver, celiac trunk, stomach, pancreas, portal vein, inferior vena cava were detected and he died of the primary disease at the 18th month of diagnosis. The third patient, a girl with unfavorable outcome (patient #10) had signs of severe virilization and a very high T level (785 ng/dL) prior to surgery and was followed by T levels and imaging studies postoperatively. At the 17th postoperative month, T level was 61.6 ng/dL, MR imaging was normal, but at 23rd month, the T level increased to 123 ng/dL and a recurrent mass of 3x2 cm at the adrenal lodge with hepatic and pulmonary metastases appeared on imaging. Mitotane combined with cisplatin and etoposide were started, but the lesions progressed and she was lost to follow-up one year after her recurrence.

Three patients (patient #12,17,18) were considered inoperable at the time of admission. Patient #12 had a huge adrenal mass with multiple pulmonary metastases. He was started on mitotane; however, he did not come back for the follow-up visit. Patient #17 had a big mass that had infiltrated the kidney and the liver, so it was decided not to operate. Cisplatin, mitotane therapies were started. Ketoconazole was also administered to decrease the very high cortisol levels promptly. Patient #18 had both hepatoblastoma and ACT simultaneously suggesting a hereditary cancer syndrome, probably Li Fraumeni syndrome. Chemotherapy has been the primary choice in this patient. 

## DISCUSSION

ACTs in childhood differ from adult type ACTs in that they are generally hormonally active. Also, they have a tendency to appear in prepubertal ages (8,9). Excess hormones are expected to simplify the diagnosis of ACT in this age group since clinical findings of excess adrenal hormones draw attention and can easily be noticed before puberty. Thus, early diagnosis may be one reason for better prognosis of the disease in children in comparison to adults. Most of our patients with signs of virilization were prepubertal, thus it had been easier to notice the associated abnormal hair growth. The clinical picture produced by excess androgens due to ACT can easily be masked during puberty and adulthood in men and makes the disease difficult to recognize. One of our patients at ten years of age (patient #17), who had Tanner stage 4 pubic hair development, was easily ignored by the family. 

Evaluation of the hormone profile in pediatric ACT is crucial both at the time of diagnosis and during follow-up. A good example is cortisol-secreting ACTs. In some cases, the clinical findings of hypercortisolism may not be overt, but the excess cortisol from the tumor may be suppressing the normal adrenal cortical tissue bilaterally. In such cases, excision of the tumor may lead to signs of adrenal insufficiency or crisis during surgery or after surgery during stress since adrenal glucocorticoid production may not recover quickly after tumor removal. Thus, any requirement of steroid coverage should be evaluated by analysis of hormone profile before surgery. Pretreatment endocrine evaluation also helps detect abnormally elevated adrenocortical hormones which are tumor markers that can be used as indicators of recurrence during follow-up.

Data regarding ACT during childhood is scarce; however, it was stated that in childhood, the most prevalent form is virilizing ACT (10,11,12,13,14,15,16). Michalkiewickz et al (13) investigated the data of 254 children with ACT from an international registry and suggested that 55.1% of children with ACT had a tumor leading to isolated virilization and 29.2% had a tumor leading to combined virilization and Cushing’s syndrome. That study did not publish any data related to hormone levels. Similarly, Ciftci et al (12) stated that virilization alone was seen in 40% of patients and virilization accompanied with Cushing’s findings was seen only in 10% of patients. Teinturier et al (14), in their retrospectively analyzed series of 54 patients, reported that 40% of their cases showed isolated androgen excess, whereas hyperandrogenism accompanied by hypercortisolism was observed in 25% of the tumors. One constraint of that study is that complete hormonal workup was not carried out in all patients. In a recent paper from China, hormone status was analyzed in 34 children with ACT in more detail; however endocrinological evaluation was not complete in all patients (15).

In the current analysis, nearly 38.8% of the patients were admitted with clinical findings suggesting isolated virilization, whereas 27.7% had signs attributable to both virilization and Cushing’s syndrome at the time of admission. This is concurrent with previous reports suggesting that most ACTs secrete isolated androgens and some - a combination of androgens and glucocorticoids. However, hormonal workup of the current series reveals that this is not the case. Hormonal workup revealed that half of the patient population (55.5%) had tumors secreting a combination of androgens and cortisol, whereas only 27.7% had ACTs secreting androgens alone. Interestingly, 42.8% of children with clinical signs of virilization alone had elevated cortisol levels and 2/3 of children who seemed to have isolated Cushing’s syndrome clinically had elevated androgen levels in addition to excess glucocorticoids. Thus, complete hormonal evaluation is required in all patients with childhood ACTs to determine activity of the tumor definitively. This approach yields two beneficial results. First of all, it helps specify which hormone/hormones are secreted so that these data may be used in the follow-up as a reliable tumor marker. The second benefit is for patients who have excess cortisol secretion without any sign of Cushing’s syndrome as yet. Since hypercortisolism suppresses normal adrenocortical tissue and eventually leads to adrenal insufficiency and crisis especially after surgical removal of the tumor, a diagnosis of “silent” hypercortisolism may help protect the patient from adrenal insufficiency.

In the current series, tumor recurrence in two patients was detected by increased hormone levels before the tumor became detectable by imaging techniques. Also, 60% of the patients had postoperative adrenal suppression which could last as long as 24 months. Hypercortisolism may exist even in patients with basal cortisol levels within the normal range. Thus, either steroid coverage should be routine in every patient undergoing operation for tumor removal (and/or adrenalectomy) or further evaluation using early morning-late evening (08.00-20.00) samples for diurnal rhythm of cortisol secretion and/or 24-hour urinary free cortisol level should become routine preoperatively. Chen et al (15) also showed a higher frequency of disturbance in circadian rhythm of cortisol secretion, while elevation of basal cortisol level was less common among patients. Thus, it is imperative to assess cortisol over secretion in patients with ACT thoroughly even if basal cortisol levels are normal or clinical signs of hypercortisolism do not exist.

Hyperandrogenism is the most frequent hormonal abnormality in children with ACTs. In our series, 15 (88%) of 17 children with functional ACTs had hyperandrogenism. DHEA and DHEA-S are the major androgens secreted from the adrenal cortex (9,10). It is interesting to observe that 40% (6/15) of patients with hyperandrogenism had very low DHEA-S levels. Three fourths of patients with hyperandrogenism had elevated T levels characteristically exceeding 100 ng/dL. Even a very high AS can be the tumor marker of ACTs. So, ACTs secreting excess androgens may vary in their secretion and a high DHEA-S is not a rule for diagnosis. It is essential to measure various androgens secreted from the adrenals including T, DHEA-S, AS and 17(OH)P. 

In conclusion, endocrinologic workup at the time of admission as well as during and after surgery for ACTs is crucial especially in childhood. All hormones secreted from the adrenal cortex should be measured in every patient with an ACT. Limited laboratory evaluation with selected hormones suggested by clinical findings would fail to identify all tumor markers especially in functional tumors that are clinically silent. The majority of ACTs in childhood secrete a combination of androgens and glucocorticoids. Moreover, androgen secretion may vary from one tumor to another, excess DHEA-S may not be the hallmark in all tumors and T exceeding 100 ng/dL is a common finding. Determination of hormonal secretion from the tumor not only provides a valuable tool for the follow-up as a tumor marker, but also helps determine the risk of intra- and postoperative adrenal suppression. 

## Figures and Tables

**Table 1:**
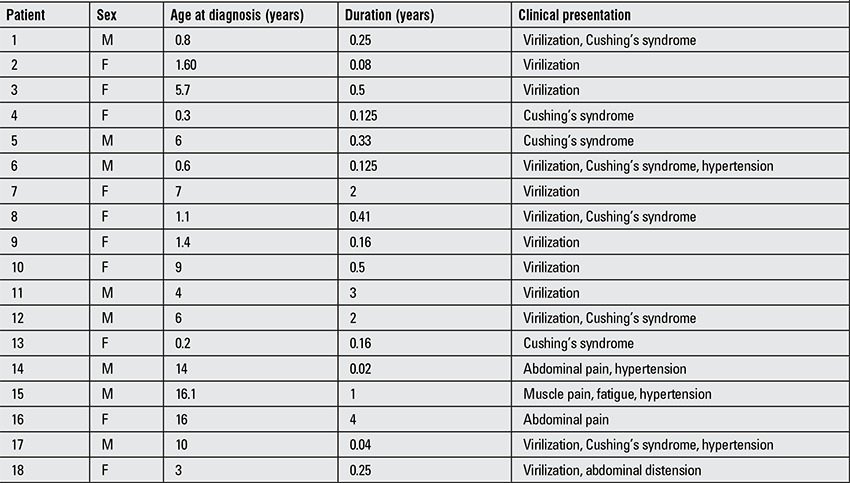
Clinical characteristics of the patients

**Table 2:**
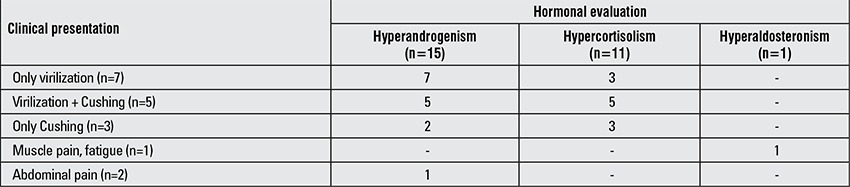
Laboratory findings as related to the clinical findings in the patients in this series

**Table 3:**
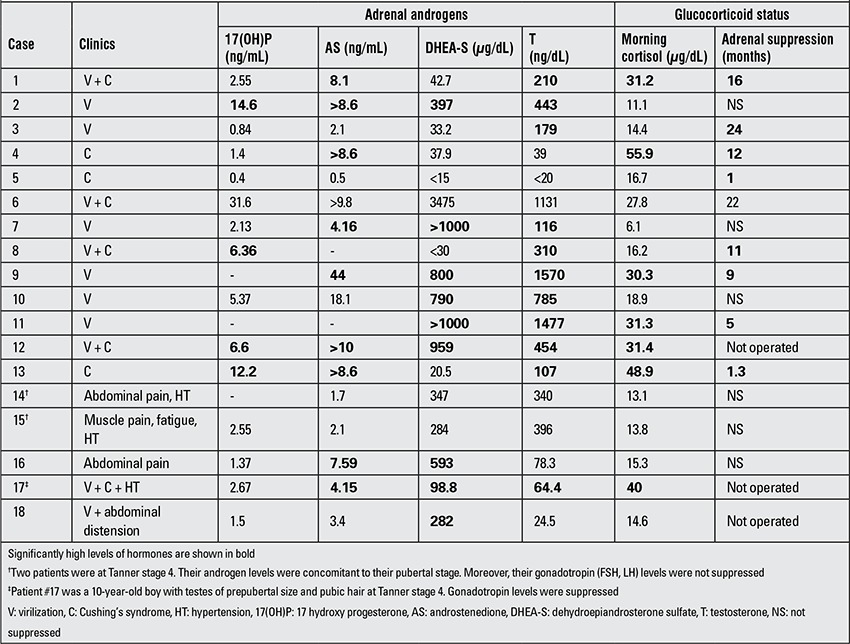
Adrenal androgen levels and glucocorticoid status of the patients
